# NEUROPROTECTIVE EFFECT OF APOE2: EVIDENCE AND IMPLICATION FOR COGNITIVE AGING

**DOI:** 10.1093/geroni/igz038.2312

**Published:** 2019-11-08

**Authors:** Paola Sebastiani, Nalini Raghavachari

**Affiliations:** 1 Boston University, Department of Biostatistics, Boston, Massachusetts, United States; 2 National Institutes of Health, Bethesda, Maryland, United States

## Abstract

Apolipoprotein E is a glycoprotein mediator and regulator of lipid transport and uptake. The APOE4 allele has been associated with higher risk of Alzheimer’s disease and of mortality, but the protective effect of the less prevalent APOE2 allele is less well established. This symposium will bring together experts in epidemiology, molecular biology, neurology and systems biology to clarify the role of APOE2 in longevity and cognitive health, to describe molecular targets of APOE alleles, and to suggest mechanisms of protection conferred by APOE2 using IPSc derived systems. Dr Seshadri will show the strong association between APOE2 and human longevity using data of 38,537 individuals of European ancestry. Mr Sweigart will examine longitudinal trajectories of cognitive function in participants of the Long Life Family Study and the New England Centenarian Study and show that carriers of the homozygote genotype of APOE2 have a significant slower rate of decline of their cognitive function compared to carriers of other genotypes. Dr Monti will present an association analysis of APOE genotype data with 4137 human proteins in serum of 222 New England Centenarian Study participants. The analysis discovered a signature of 16 proteins that associated with different APOE genotypes, and replicated in 3 independent studies. Dr. Ellerby will summarize her recent analyses that used transcription analysis of isogenic iPSCs with APOE2 and APOE4 homozygote genotypes differentiated into inhibitory GABAergic neurons to show that E2 inhibitory GABAergic neurons regulate genes involved in nuclear division, DNA integrity and DNA damage checkpoint.

